# Electrophysiological correlates of presuppositional inference in contrastive focus

**DOI:** 10.3389/fnhum.2026.1905786

**Published:** 2026-09-08

**Authors:** Zhiqiang Yang, Hongshuang Nie, Kevin B. Paterson, Sixiao Li, Lijing Chen

**Affiliations:** 1 School of Psychology, Fujian Normal University, Fuzhou, China; 2 School of Ethnology and Sociology, Southwest Minzu University, Chengdu, China; 3 College of Sociology and History, Fujian Normal University, Fuzhou, China; 4 School of Psychology and Vision Sciences, University of Leicester, Leicester, United Kingdom

**Keywords:** ERPs, it-cleft, only, pragmatic inference, presupposition

## Abstract

This study investigated the electrophysiological correlates of presuppositional inferences in contrastive focus during online reading. In a series of mini-discourses involving two characters, contrastive focus was marked by either the Chinese focus particle *zhiyou* (equivalent to *only*) or *shi* (equivalent to an *it*-cleft), which carry different presuppositions. Before the focus sentence, a critical context sentence was presented that was congruent with the *zhiyou*-presupposition (i.e., the two characters previously did the action together) or with the *shi*-presupposition (i.e., the other character previously did it alone). Participants read and listened to the discourses alongside a partner, as social context may potentially facilitate pragmatic inference. Results showed that the particle *zhiyou* elicited a larger LPC waveform following incongruent than congruent contexts, while the discourse-final word with *shi*-focus elicited a larger P300 following incongruent than congruent contexts. These findings suggest that presupposition violations of *only*-focus relate to early disconfirmation of pragmatic expectations, while presupposition violations of cleft-focus involve late-stage pragmatic integration during discourse update. These results highlight the role of focus particles in modulating presuppositional inference and offer insights into their distinct electrophysiological signatures.

## 1 Introduction

Successful conversation relies on participants understanding each other’s conversational implicatures, which requires pragmatic inferences by the comprehender (Grice, 1975; Kravtchenko and Demberg, 2022). These implicatures are often cued linguistically (Bohn et al., 2022; Chen and Yang, 2015; Deckert et al., 2021; Paterson et al., 2007). For example, in the sentence “Only John attended the meeting,” the focus particle *only* conveys the contrastive inference that others did not attended (Ni, 1996; Sedivy, 2002; Umbach, 2004). A similar contrast is cued by the *it*-cleft construction in “It was John who attended the meeting” (Hoeks et al., 2023). In Chinese, the focus particle *shi* (是) functions similarly to *it*-clefts, while *zhiyou* (只有) functions like *only* (Chen et al., 2012; Chen et al., 2019). The two expressions differ fundamentally in their grammatical and pragmatic functions. *Shi* is polysemous, serving as either a copula or, as in the present study, a focus marker. In its focus-marking use, *shi* gives prominence to the focused constituent and signals a contrast with contextually relevant alternatives, while its removal does not render the sentence ungrammatical (Fang, 1995; Chen et al., 2014). *Zhiyou*, by contrast, is a scope adverb associates with the focused constituent that constrains the alternative set, corresponding closely to the restrictive meaning of English *only* (Umbach, 2004; Chen et al., 2019). Thus, although both forms establish contrastive focus, they do so through different grammatical mechanisms and impose different constraints on discourse representation. The focus particle *zhiyou* presupposes that the focused individual and at least one other person were expected to perform the action together (Umbach, 2004). By contrast, *shi* presupposes that someone other than the focused individual was expected to perform it. How these distinct presuppositions are processed, and what their electrophysiological correlates are, remains unknown.

Event-related potential (ERP) studies on contrastive focus have primarily examined how the brain responds when the focus structure is violated, without directly manipulating presuppositions. For *only*-focus, Stolterfoht et al. (2007) investigated the German particle *nur* (*only*) by contrasting sentences with and without the particle (e.g., “the principal criticized [only]/[] the pupil, and the principal did not criticize the teacher”). When *nur* was omitted, the subsequent alternative noun (e.g., “the teacher”) elicited an enhanced late positive component (LPC) between 350 and 1,300 ms, interpreted as reflecting reanalysis and revision processes. Drenhaus et al. (2011) used *nur* to create a mismatch between the focus structure and a later phrase (e.g., “Only Mary can play the piano, and, besides, the violin/*Luise and Jana…”). The incongruent phrase “Luise and Jana” elicited an enhanced LPC between 600 and 800 ms over centro-parietal electrodes, again reflecting reanalysis. Notably, in both studies the effect was observed on words following the focused element, leaving open the question of whether a presupposition violation could be detected earlier, at the focus particle or the focused word itself.

For cleft-focus and its Chinese counterpart, *shi*-focus, two ERP components have been implicated. First, an enhanced N400, a negativity peaking around 400 ms and associated with semantic/pragmatic integration difficulty (Kutas and Federmeier, 2011; Kutas and Hillyard, 1980), has been observed at the focused word or subsequent critical phrase. Cowles et al. (2007) presented cleft sentences as answers to wh-questions and found that an inappropriate focused word (e.g., answering “It was the queen that silenced the banker” to the question “Who did the queen silence…?”) elicited an enhanced N400 between 200 and 500 ms post-stimulus onset. Similarly, when Drenhaus et al. (2011) replaced *nur* with a cleft construction, the mismatched alternative phrase elicited an N400-like negativity between 400 and 600 ms. In summary, focus structure violations can elicit an enhanced N400 effect at the focused element/subsequent critical alternative phrase. Second, an enhanced P300, a positivity peaking around 300 ms, has been consistently reported at the sentence-final word when pragmatic integration is difficult (Chen, 2018; Lin et al., 2025). Chen (2018) examined Chinese *shi*-focus and found that when the prior context lacked an overt alternative for the focused character (e.g., “When Ling Pan was shopping, it was Ling Pan who bought a garment”), the discourse-final word elicited an enhanced P300 between 265 and 495 ms. Lin et al. (2025) replicated this finding: when the context provided an inappropriate alternative, the sentence-final word elicited an enhanced P300 between 225 and 700 ms. These P300 effects are thought to reflect contextual integration demands (Chen, 2018; Cowles et al., 2007). Together, these studies show that cleft-focus violations can evoke an enhanced N400 on the focused element and/or an enhanced P300 at the discourse-final word, depending on the locus of the integration challenge. Critically, however, these ERP studies manipulated the availability or appropriateness of alternatives, or, as in Cowles et al. (2007), the congruence between the question and the cleft response—but not the background presupposition. Whether presupposition violations elicit similar ERP components, and at which word(s) in the sentence, remains an open question.

To derive precise predictions, we analyzed the presuppositions of *zhiyou* and *shi* in greater depth. For *zhiyou*-focus, the presupposition requires at least two individuals, including the focused one (Umbach, 2004). If the context introduces only a single individual, the violation is numerical: the context creates an expectation that the following text will not contain *zhiyou*, because its presupposition of “at least two” conflicts with the actual context. If readers actively anticipate upcoming linguistic structure based on context, a presupposition violation could be detected at *zhiyou* itself. By contrast, *shi*-focus merely presupposes that someone other than the focused individual was expected (Umbach, 2004); the exact number of presupposed individuals is not decisive. Determining a violation requires knowing the identity of the focused individual. Hence, the earliest point at which the violation can be detected is at the focused name, or later at the sentence-final word. Based on this reasoning, we hypothesized that incongruent contexts would elicit an enhanced LPC on the focus particle *zhiyou* early in processing, whereas for *shi*-focus the effect would appear on the focused name (possibly an N400) or the discourse-final word (possibly a P300).

However, directly testing these predictions with ERPs presents a methodological challenge. Previous studies from our lab suggest that the effect of presupposition violation is sensitive to the number of trials. In a self-paced reading experiment (Li, 2021, master’s thesis), reading time increased for presupposition violations when only the first 48 trials were analyzed; the effect disappeared when all 144 trials were analyzed, indicating that participants might adapt to presupposition violation as trials accumulated, consistent with previous studies on expectation adaptation of anomalous language structures (Fine et al., 2013; Hoek et al., 2021). Moreover, two ERP experiments from our lab using a large number of trials (144 and 128 trials, respectively) both failed to detect reliable presupposition-related effects (Li, 2021, master’s thesis; Yang, 2024, master’s thesis). Given that ERP analysis typically requires a minimum of about 30 trials per condition to ensure an adequate signal-to-noise ratio, simply reducing trial counts is not feasible. The core challenge, therefore, is to sustain participants’ pre-suppositional processing across a sufficient number of trials so that its electrophysiological correlates can be captured.

We addressed this challenge by introducing a social partner during the reading task. Social facilitation theory proposes that the mere presence of another person can heighten attentional arousal and improve performance on tasks (Zajonc, 1965). In the domain of language comprehension, reading with a partner has been shown to enhance perspective-taking and increase neural sensitivity to semantic anomalies (Forgács et al., 2022; Jiménez-Ortega et al., 2022; Jouravlev et al., 2019; Rueschemeyer et al., 2015; Westley et al., 2017). Given that pragmatic inference typically requires recognizing a speaker’s intention and engages the perspective-taking system, we reasoned that a partner’s presence, by boosting perspective-taking, may strengthen presuppositional inferences for contrastive focus, making it robust enough to be sustained across the large number of trials required for ERP recording.

We therefore designed an ERP experiment using three-sentence mini-discourses (Table 1). The first sentence introduced two characters by name. The second sentence established either a one-character context (congruent with *shi*’s presupposition) or a two-character context in which the characters acted together (congruent with *zhiyou*’s presupposition). The third sentence assigned focus to one character using either *shi* or *zhiyou*, creating congruent and incongruent conditions. The mini-discourses were constructed to establish a shared discourse representation with two potential referents before manipulating which referent(s) had previously performed the relevant action. This allowed the key context sentence (the second sentence) to establish the alternative set against which the presuppositions of *zhiyou*-focus and *shi*-focus could be evaluated, while the target sentence (the third sentence) introduced contrastive focus within an ongoing discourse rather than in an isolated sentence. The paradigm therefore provided the discourse structure required for pragmatic focus processing while allowing the critical presuppositional relation between context and focus construction to be manipulated experimentally. Critically, to ensure deep processing of the contextual information while maintaining the partner’s presence, the first and third sentences were presented visually to both the participant and the partner, whereas the second sentence was delivered via earphones to the participant (Mariadassou et al., 2023), so that the partner remained unaware of the key contextual content (Jouravlev et al., 2019; Rueschemeyer et al., 2015). EEG was recorded simultaneously from both individuals using a dual-EEG setup (Sebanz and Knoblich, 2021; Wang et al., 2020).

**Table 1:** Example discourse illustrating the four experimental conditions.

Conditions	Background sentence	Key context (audio sequence)	Target sentence
One-character context/*shi*-focus	美玲和建辉开了家书店,	上个月美玲采购图书, 但	这个月/*是*/建辉/采购/图书。
	Meiling and Jianhui run a bookstore,	Last month Meiling purchased books, but	This month/*shi*/Jianhui/purchased/books.
	Meiling and Jianhui run a bookstore.	Last month Meiling purchased (the) books. But	This month/*it was*/Jianhui**/***(who)* purchased/(the) books.
One-character context/*zhiyou*-focus	美玲和建辉开了家书店,	上个月美玲采购图书, 但	这个月/*只有*/建辉/采购/图书。
	Meiling and Jianhui run a bookstore,	Last month Meiling purchased books, but	This month/*zhiyou*/Jianhui/purchased/books.
	Meiling and Jianhui run a bookstore.	Last month Meiling purchased (the) books. But	This month/*only*/Jianhui/purchased/(the) books.
Two-character context|*shi*-focus	美玲和建辉开了家书店,	上个月他俩一起采购图书, 但	这个月/*是*/建辉/采购/图书。
	Meiling and Jianhui run a bookstore,	Last month they together purchased books, but	This month/*shi*/Jianhui/purchased/books.
	Meiling and Jianhui run a bookstore.	Last month they purchased (the) books together. But	This month/*it was*/Jianhui**/***(who)* purchased/(the) books.
Two-character context|*zhiyou*-focus	美玲和建辉开了家书店,	上个月他俩一起采购图书, 但	这个月/只有/建辉/采购/图书。
	Meiling and Jianhui run a bookstore,	Last month they together purchased books, but	This month/*zhiyou*/Jianhui/purchased/books.
	Meiling and Jianhui run a bookstore.	Last month they purchased (the) books together. But	This month/*only*/Jianhui/purchased/(the) books.

For each condition, the original text is presented on the first line; the second and third lines show the word-for-word translation and the holistic translation, respectively. Focus particles are shown in italics and focused names are underlined. Slash lines in the target sentence indicate the segments for presentation to participants. Words in parentheses are added for translation purposes and do not exist in the original Chinese versions. The text was presented without italics, underlining or slashes in the experiment.

We anticipated that, if the partner’s presence successfully enhanced presuppositional inference, incongruent contexts would elicit distinct ERP effects depending on focus type. For *zhiyou*-focus, an incongruent one-character context should elicit an enhanced LPC effect (Drenhaus et al., 2011; Stolterfoht et al., 2007) on the focus particle *zhiyou*. For *shi*-focus, an incongruent two-character context should elicit an enhanced N400 effect (Cowles et al., 2007; Kutas and Federmeier, 2011) on the focused name, and/or an enhanced P300 effect (Chen, 2018; Lin et al., 2025) on the sentence-final word.

## 2 Methods

### 2.1 Participants

G*Power 3.1 was used to estimate the required sample size based on an effect size of *f* = 0.25, *α* = 0.05, and statistical power of 0.80. This indicated that 24 participants, each paired with a partner, would be needed (48 individuals in total). To allow for possible data loss, a total of 56 undergraduate and graduate students (age range: 17–24 years; mean age: 19.5 years; 34 females; 54 right-handed) were recruited for the experiment. All were native Chinese speakers with normal or corrected-to-normal vision, and none reported any history of brain injury or reading difficulties. Upon arrival at the laboratory, they were randomly assigned in pairs to either the participant role (who heard the key context sentence) or the partner role (who did not hear it). As a result, 28 students (age range: 17–24 years; mean age: 19 years; 17 females; all right-handed) were assigned to the participant role, while the remaining 28 were assigned to the partner role. All of them gave written informed consent and were paid ¥60 for participating. The experiment was approved by the Institutional Ethics Review Board of School of Psychology at Fujian Normal University.

### 2.2 Stimuli

The experimental materials consisted of 128 sets of three-sentence discourses (27–46 Chinese characters in length, mean = 34 characters). Each set of discourses had four versions. The first sentence of each discourse introduced the story background and two characters whose genders either matched or differed (e.g., both male, both female, or one male and one female). The second sentence (the key context) described a past routine event involving either one of the two characters (one-character condition) or both of them (two-character condition). In the third sentence, the target character (i.e., the one not mentioned in the second sentence in the one-character context) was described as performing the routine event this time, and was focused using *shi* (cleft-focus condition) or *zhiyou* (*only*-focus condition). The anaphora of focused name was nearly balanced by gender and mentioned location^1^. The second and third sentences were linked by either the adversative conjunction *dan* (meaning *but*, 90 sets of materials), *buguo* (marking a milder adversative relation than *but*, 24 sets of materials), or no conjunction at all (14 sets of materials).

The fluency of the experimental discourses was evaluated by 28 participants who did not participate in the formal experiment, using a five-point scale (1 = “not fluent,” 5 = “fluent”). An interaction between context and focus was observed [*F*(1,27) = 71.81, *p* < 0.001, *η_p_^2^* = 0.73]. Discourses with cleft-focus scored higher for fluency when the context involved a one-character event (M = 4.07, SD = 0.64) than a two-character event (*M* = 3.74, SD = 0.94; *F*(1,27) = 4.54, *p* < 0.042, *η_p_^2^* = 0.14), while fluency scored higher for discourses with *only*-focus when the context involved a two-character (M = 4.52, SD = 0.45) than a one-character event (*M* = 2.59, SD = 0.84; *F*(1,27) = 127.17, *p* < 0.001, *η_p_^2^* = 0.83).

An additional 128 filler discourses were created. These were of similar construction to the experimental items, with varying numbers of discourse characters (0, 1, or 2) and different types of conjunctions. Ten additional practice discourses of similar construction were also created. For all discourses, the second sentence and the conjunction at the beginning of the third sentence (when present) were converted into speech in a female voice using *Tick Dub* software (for the experimental discourses: recording duration ranging from 1,617 to 4,137 ms; Mean = 2,772 ms; for the filler discourses: recording duration ranging from 969 to 4,317 ms; Mean = 2,189 ms).

Four counterbalanced lists were created, each containing 266 discourses, including 128 experimental discourses (32 discourses per condition), 128 filler discourses and 10 practice discourses. Each list was assigned randomly to 7 participants.

### 2.3 Procedure

Each participant and their partner were seated in a soundproof room. The participant wore earphones whereas the partner did not. In half of the pairs, the participant sat on the left side of the screen and the partner on the right side; this arrangement was reversed for the other half. Text was presented on the monitor using a 40-point Courier New font (monitor resolution: 1920 × 1,080); and audio sequences were delivered via the earphones.

**Figure 1:**
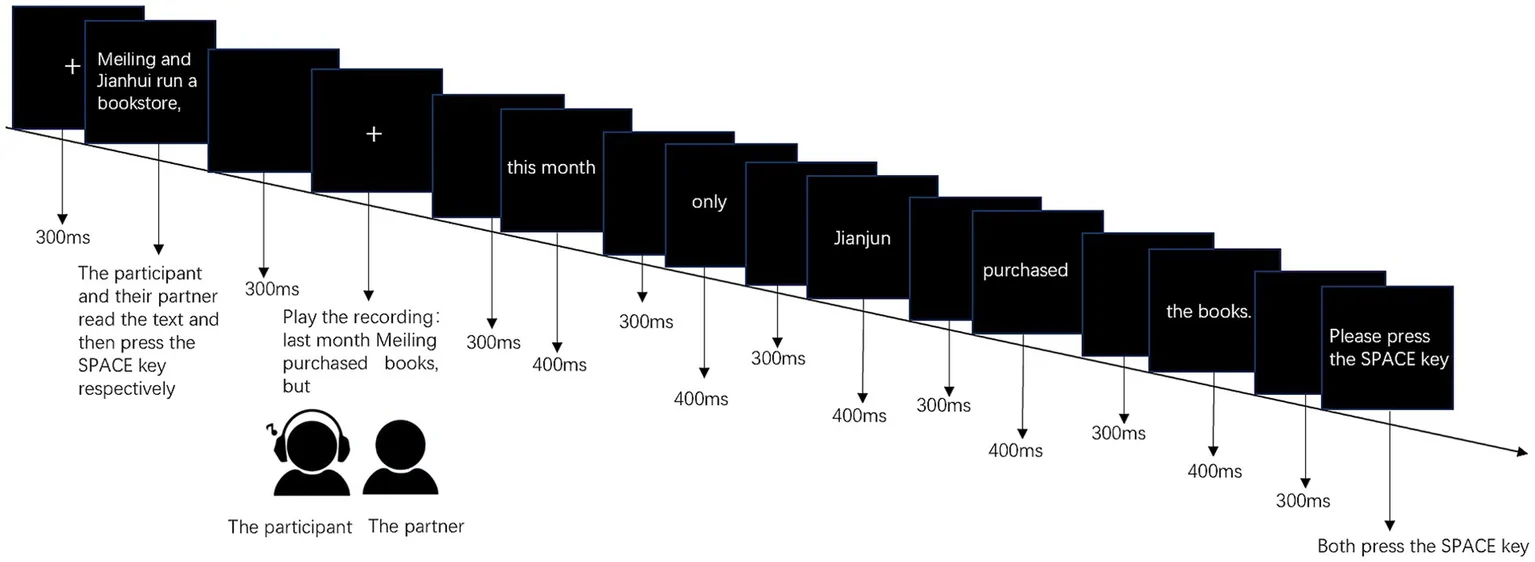
Experimental flowchart.

At the beginning of each trial, a “+” was displayed at the screen center for 300 millisecond, followed by a 300-ms interval. Then the first (background) sentence was displayed. The participant and the partner each pressed the space bar upon finishing reading it. Then a “+” was displayed at screen center. After 300 ms, the audio sequence was presented to the participant via the earphones, while the partner fixated the “+.” Once the recording ended, the “+” disappeared. After another 300 ms, the final (target) sentence was presented segment-by-segment. Each segment, consisting of a single word or a short phrase, was displayed for 400 ms, with a 300-ms interval between segments. This presentation rate supports fluent reading and has been commonly used in prior studies of focus processing (Chen, 2018; Lin et al., 2025). The sentence-final period was presented together with the final word. About one third of trials were followed by a yes/no comprehension question. In these trials, the person seated on the left side used the “A” key to indicate a correct response and the “S” key to indicate an incorrect response, whereas the person on the right side used the “K” key to indicate a correct response and the “L” key to indicate an incorrect response. For the remaining trials, both pressed the space bar to continue. The next trial began once both had responded (Figure 1).

Trials were presented in 8 blocks, each with 32 trials displayed in pseudo-random order. Participants and their partners were given short breaks between blocks. The entire experiment lasted about 3.5 h, including preparation time.

### 2.4 EEG recording and pre-processing

EEG was recorded from all participants and partners using 32 Ag/AgCl electrodes (Neuroscan EEG detection Australia -GRAEL 32 CHANNEL NEONET), with a sampling rate of 1,024 Hz. Vertical and horizontal eye movements were recorded using bipolar electrodes above and below the left eye and on the outer side of both eyes. Impedances were kept below 5KΩ for all electrodes.

Offline, only data from the participant’s role were analyzed. Data from two participants were excluded for comprehension accuracy below 80%. Then, EEG data were re-referenced to the average of bilateral mastoids using the EEGLAB (v2022.1, Delorme and Makeig, 2004) and ERPLAB toolboxes (7.0.0, Lopez-Calderon and Luck, 2014). Data were filtered using a 0.05 Hz-40 Hz bandpass filter. And ICA was used to remove eye blink artifacts. Data were segmented into epochs spanning from −100 ms to 700 ms relative to the presentation of critical stimulus (the focus particle, focused name, and discourse-final word), with a 100-ms pre-stimulus baseline. Trials with voltage exceeding ±75 μV were rejected. Two participants’ data were excluded from the further analysis due to high data rejection rate (>35%). Average artifact rejection was 7.47% for the remaining participants.

### 2.5 Data analysis

Time windows of interest were identified based on grand-average ERP waveforms and previous research (Kutas and Federmeier, 2011; Lin et al., 2025; Stolterfoht et al., 2007). Three time windows were chosen for analysis: 350–700 ms (LPC) for the focus particle, 300–500 ms (N400) for the focused name, and 300–700 ms (P300) for the discourse-final word. For each window, repeated-measure analyses of variance (ANOVA) were conducted for signals averaged across electrodes groups.

The electrodes were grouped and averaged in five regions: central (CZ, CPZ), left anterior (F3, FC3), right anterior (F4, FC4), left posterior (P3, CP3) and right posterior (P4, CP4). For each time window, a three-way ANOVA with the factors context (one-character, two-character), focus (cleft-focus, *only*-focus), and brain region (left anterior, right anterior, central, left posterior, right posterior) were computed. Significance was set at *α* = 0.05. A Greenhouse and Geisser (1959) correction was applied for dfs > 1.

## 3 Results

### 3.1 Behavioral results

The mean comprehension accuracy was 92.96%, indicating that participants successfully processed and comprehended the materials.

### 3.2 ERP results

**Figure 2:**
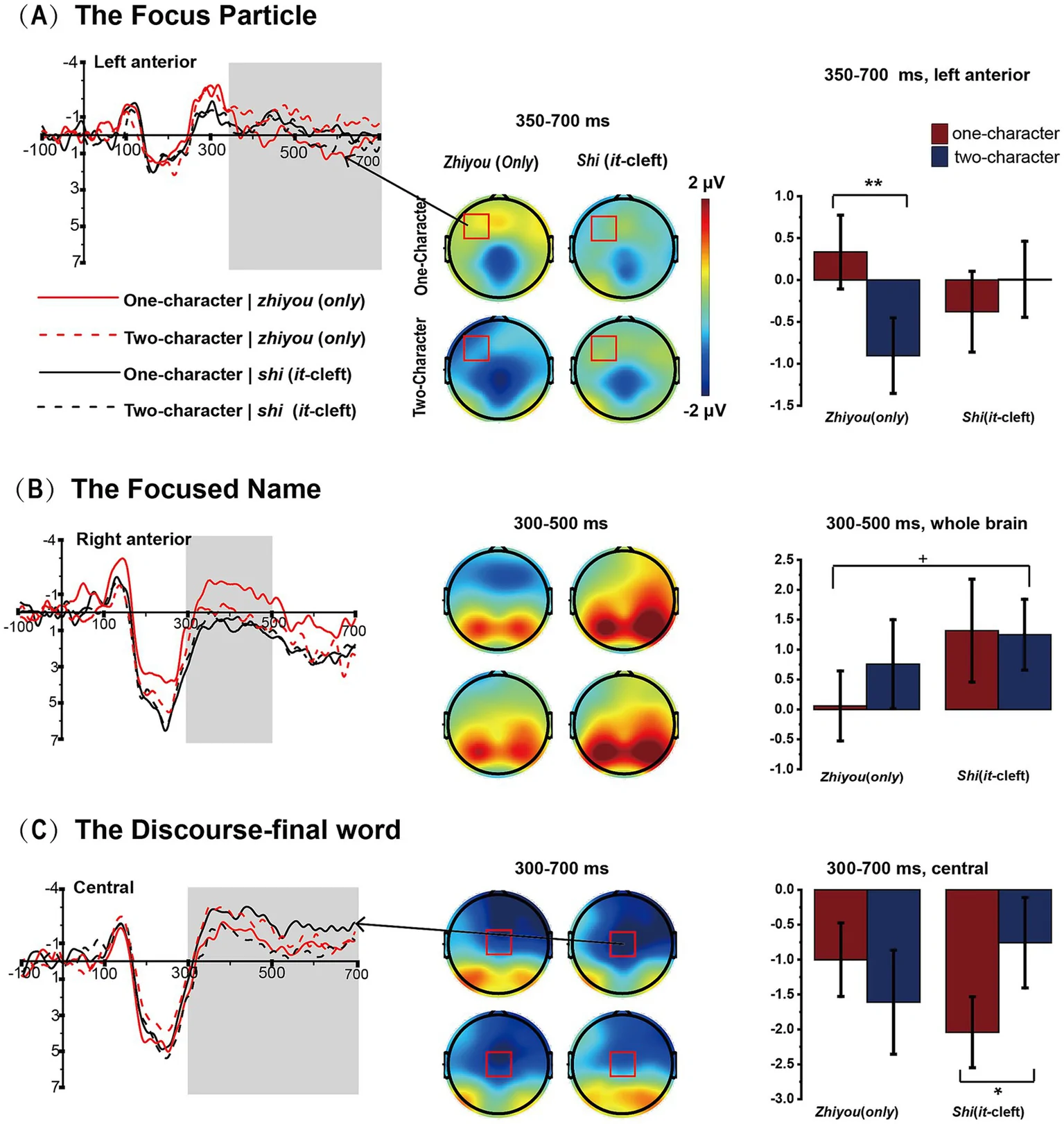
Grand-average ERPs, topographic maps, and bar graphs for **(A)** focus particles, **(B)** focused names, and **(C)** discourse-final words, shown for the representative brain regions and time windows in each of the four conditions. In the ERP plots, gray bars indicate the time windows for analysis. In the topographies, red diamonds indicate the representative brain regions displayed in the ERP graphs. In the bar graphs, the histograms represent the mean voltage, and the error bars indicate the SEM. * Denotes a significant difference between two context conditions for *shi*-focus (*p* < 0.05). ** Denotes a significant difference between two context conditions for *zhiyou*-focus (*p* < 0.01). + Denotes a significant difference between *zhiyou*-focus and *shi*-focus (*p* < 0.05).

Figure 2 shows average waveforms elicited by the focus particles, focused names, and discourse-final words for the representative regions. Table 2 presents the statistical results for the mean ERP amplitudes in each window.

**Table 2:** Statistical results for the mean ERP amplitudes elicited by the three types of critical words.

Factors	Focus particle			Focused name			Discourse-final word		
	350–700 ms			300–500 ms			300–700 ms		
	*F*	*p*	η*_p_^2^*	*F*	*p*	η*_p_^2^*	*F*	*p*	η*_p_^2^*
Context	0.788	0.384		0.629	0.436		0.464	0.503	
Focus	0.595	0.449		6.427	0.019	0.218	0.002	0.968	
Context * focus	1.244	0.276		0.889	0.355		3.257	0.084	
Context * region	0.331	0.776		0.618	0.599		0.222	0.809	
Focus * region	0.933	0.427		1.913	0.148		0.395	0.700	
Context * focus * region	3.203	0.032	0.122	2.000	0.135		3.114	0.019	0.119

*n* = 24.

#### 3.2.1 Focus particle

In the 350–700 ms (LPC) time window, an interaction among context, focus, and brain region was observed. Further analyses showed that at the left anterior region (*F*(1, 23) = 9.86, *p* < 0.01, *η_p_^2^* = 0.30), and right anterior region (*F*(1, 23) = 8.65, *p* < 0.01, *η_p_^2^* = 0.27), *zhiyou* elicited a larger positive waveform following one-character than two-character contexts, with no corresponding effect of context for *shi* (*F*s < 1). No significant effects were observed for the other regions (*F*s < 1).

#### 3.2.2 Focused name

In the 300–500 ms (N400) time window, the analyses revealed a main effect of focus, with the *zhiyou*-focused name eliciting a more negative waveform than the *shi*-focused name. No other significant effects were observed.

#### 3.2.3 Discourse-final word

In the 300–700 ms (P300) time window, an interaction among context, focus, and brain region was observed. At the central region, for *shi*-focus, the discourse-final word elicited a larger positive waveform for the two-character than one-character context [*F*(1,23) = 4.85, *p* < 0.05, *η_p_^2^* = 0.17]; while no corresponding effect of context was observed for *zhiyou*-focus (*F* < 1.1). No significant effects were observed at the other regions (*F*s < 4.3).

## 4 Discussion

The present study investigated the electrophysiological correlates of presuppositional inference in contrastive focus. There are two main findings. First, the focus particle *zhiyou (only)* elicited an enhanced anterior LPC effect when the context was incongruent with its presupposition. Second, *shi* (cleft)-focus elicited an enhanced central P300 effect at the discourse-final word under the incongruent condition.

### 4.1 Violation of presuppositional inference elicited an enhanced LPC at the focus particle for *only*-focus

The finding that the focus particle *zhiyou* elicited an enhanced LPC in an incongruent context aligns with the hypothesis. This suggests that the presuppositional inference of *only*-focus, that the focused individual is presupposed to perform the action with others, is computed at the focus particle itself. Such a result supports the analysis that the number of presupposed referents is actively evaluated during the processing of the focus particle *zhiyou*, enabling the rapid detection of discourse incongruity. The LPC is associated with the processing of unexpected or disconfirming input during sentence and discourse comprehension (Federmeier et al., 2007), with more unexpected words eliciting larger amplitudes (Kuperberg et al., 2020). Thus, the enhanced LPC for *zhiyou* indicates that this particle was less anticipated when the context failed to provide pragmatic support for it.

### 4.2 Violation of presuppositional inference elicited an enhanced P300 at the discourse-final word for cleft-focus

For *shi*-focus, the presupposition violation elicited a P300 enhancement at the discourse-final word. Consistent with our earlier analysis, whether the presupposition was violated could not be evaluated at the focus particle. This presupposition only requires that someone other than the focused individual be expected to perform the action, without specifying the number of alternatives. Thus, whether the context violates the presupposition could not be determined until the participants had identified the focused individuals. Interestingly, the violation was not detected at the focused name either. This is likely because participants, upon reading a single focused name, could not be certain whether another focused name would follow to form a compound focus domain (e.g., “it was Jianhui and Siying who”). Consequently, the detection of the presupposition violation was plausibly delayed until the full sentence had been processed and the complete focus domain was fully determined. This delayed P300 effect likely reflects increased effort in contextual integration (e.g., Cowles et al., 2007; Chen, 2018). It aligns closely with previous studies on *shi*-focus pragmatic violations (Chen, 2018; Lin et al., 2025). Chen (2018) reported an enhanced P300 at discourse-final words for *shi*-focus sentence that lacked an explicit contrastive alternative. Similarly, Lin et al. (2025) observed P300 enhancements when integration of the focused referent was challenging due to the presence of alternative characters with different identities. Together, these findings suggest that when the discourse context is incongruent with the pragmatic inference of *shi*, readers do not identify the inconsistency locally at the focus particle or the focused name, but rather expend more effort integrating it at a later, global discourse level.

Although the LPC observed at the focus particle and the P300 observed at the discourse-final word are both positive-going components with similar latencies, they are considered distinct for several reasons. First, they differed in scalp topography (anterior vs. central). Second, they also showed distinct waveform morphologies. Third, they are associated with distinct cognitive functions. These differences support treating them as separate components (Dien et al., 2004).

### 4.3 The distinctions between *zhiyou*-focus and *shi*-focus

The present findings support the hypothesis that *zhiyou*-focus and *shi*-focus rely on distinct presuppositions, and readers are sensitive to these presuppositions. This hypothesis also accounts for results reported in previous studies. For example, Drenhaus et al. (2011) showed that when a subsequent phrase violated the exhaustivity implicature of contrastive focus, violation of *only*-focus elicited an enhanced late positivity, whereas violation of cleft-focus elicited an enhanced N400-like negativity. The authors attributed this pattern to distinct cognitive processes underlying the exhaustive meanings of two types of focus. Combining these observations with our results, we propose that because *only* uniquely specifies the focused referent, a subsequent additional referent creates an explicit logical violation, triggering reanalysis and an enhanced late positivity. By contrast, *shi* imposes weak restrictions on the number of alternatives; thus, when an extra referent is introduced, readers perceive no logical contradiction. Instead, they pragmatically integrate the additional information, which increases semantic-pragmatic integration load and elicits an enhanced N400.

In addition, the present study also found that the *shi*-focused names elicited a smaller N400 than the *zhiyou*-focused names, indicating easier semantic integration for the *shi*-focused elements than for the *zhiyou*-focused elements. This likely reflects the different functional priorities of the two particles (Chen et al., 2019). Although both give prominence to the focused elements and imply a contrast between the focused elements and their alternatives, the particle *shi* predominantly serves an emphasizing function, directing attention to the focused element and thus facilitating its integration (Chen et al., 2014), whereas *zhiyou* predominantly serves a contrastive function, which requires constructing a contrast set and thereby increases the integration load (Chen et al., 2019).

### 4.4 Pragmatic inference of contrastive focus in a social reading context

Presupposition violation effects for *zhiyou*-focus and *shi*-focus were observed at different sentential constituents, indicating distinct processing pathways for the two focus types. Specifically, violation for *zhiyou*-focus was detected at the focus particle itself. This finding extends the scope of pragmatic anticipation. Previous research has shown that pragmatic cues like focus particles can guide readers to predict upcoming semantic content (Frank and Goodman, 2012; Herbstritt and Franke, 2019; Schneider et al., 2019; Schuster and Degen, 2020). For instance, hearing *only* leads comprehenders to anticipate a restricted set of referents. The present results go a step further, showing that the pragmatic cues themselves—here, the focus particle *zhiyou*—can be anticipated based on the discourse context. This form of anticipation resembles semantic anticipation. For instance, in the one-character context, as discourse unfolds, contextual cues, including a single referent, contrastive conjunctions such as *but* (when present), and temporal contrasts, gradually constrain upcoming lexical possibilities and render the occurrence of *zhiyou* pragmatically unexpected. By contrast, in the two-character condition, the discourse context unfolds without highly constraining the possibilities of the occurrence of *shi*, so evaluation of the presupposition depends on integrating the focused referent with the broader discourse context. Consistent with this distinction, presupposition violation for *shi*-focus was observed at the discourse-final word. In line with previous studies (Chen, 2018; Lin et al., 2025), this pattern reflects the integration of pragmatic information into the discourse model. Taken together, pragmatic anticipation linked to *zhiyou* and pragmatic integration associated with *shi*-focus indicate that pragmatic inference, much like semantic processing, engages both anticipatory and integrative mechanisms. Accordingly, the two focus constructions impose different inferential demands on the comprehender. For *zhiyou*-focus, contextual information can constrain whether the focus particle itself is expected, allowing incongruity to be detected as soon as the particle is encountered. For *shi*-focus, evaluating the presupposition depends on identifying the focused referent and integrating this information with the developing discourse representation, resulting in later detection of incongruity.

The co-reading paradigm placed these processes within a socially situated discourse context. Participants and their partners shared the background and target sentences, while only the participant had access to the critical context sentence. Participants therefore evaluated the pragmatic relation between the context and focus construction while reading alongside a co-reader whose knowledge of the discourse differed from their own. Electrophysiological effects of presupposition violations were observed while participants read alongside a co-reader. This contrasts with previous studies from our lab in which participants read alone and reliable presupposition-violation effects were not observed (Li, 2021, master’s thesis; Yang, 2024, master’s thesis). Although this cross-study difference cannot establish that the presence of a social partner facilitated presuppositional inference, the present findings are consistent with this possibility. Previous research indicates that the presence of a co-reader can promote perspective-taking during language comprehension (Forgács et al., 2022; Jiménez-Ortega et al., 2022), providing a possible mechanism through which social context could support engagement in pragmatic inference. Direct evidence for such an effect would, however, require comparison of otherwise equivalent partner and no-partner conditions.

### 4.5 Limitations and future directions

There are several limitations. First, participants completed the task in the presence of a social partner, a manipulation intended to facilitate presuppositional inference. However, because the present study did not include a no-partner condition, the contribution of social presence to the observed effects cannot be established directly. Moreover, the auditory presentation of the context sentences may also have influenced pragmatic processing, and the contributions of these factors cannot be disentangled in the present design. Researchers interested in this issue may examine their effects by orthogonally manipulating these two factors. Second, the impact of a social partner may be modulated by individual differences in sociality (Uziel, 2007). Moreover, the exact mechanism through which the presence of a social partner modulates neural processing remains unresolved. Follow-up work could measure sociality as a covariate to clarify how it influences pragmatic inference within social contexts. Third, while the segment presentation rate used in the experiment is commonly used in comparable ERP studies, it is comparatively slow (400 ms per word plus a 300 ms ISI, yielding a total of 700 ms processing time per word) relative to natural conversation. This extra processing time may have facilitated pragmatic inference-making. Future studies could examine whether similar effects are obtained under more naturalistic temporal constraints. Fourth, the present study adopted a classic co-reading paradigm (Forgács et al., 2022; Rueschemeyer et al., 2015), in which stimuli were presented partly visually and partly auditorily. Future studies could present stimuli fully auditorily, which may improve the ecological validity of the observed effects. Finally, whereas the present study focused on ERP signatures of presuppositional inferences, further investigations may adopt time-frequency analysis to supply complementary evidence and advance understanding of the underlying neural mechanisms. Addressing these questions will contribute to a more comprehensive understanding of how contextual, social, and temporal factors collectively shape presuppositional inference in real time.

## 5 Conclusion

In conclusion, the present study reveals a dissociation: the presupposition violation of *zhiyou*-focus elicits a disconfirmation of predictions at the focus particle, reflected in an enhanced LPC, whereas the presupposition violation of *shi*-focus leads to a delayed integration effort at the discourse-final word, reflected in an enhanced P300. This dissociation arises from a fundamental difference in when presupposition violations can be detected for the two focus types. That is, for *zhiyou*-focus, the numerical violation is detectable immediately at the focus particle, whereas for *shi*-focus, detection must await the identification of the focused individual.

## Data Availability

The data and materials are available at https://osf.io/g27bv/overview?view_only=0779962b6c69463fb733f1108453d4f1.

## Footnotes

1 The focused name referred to the first-mentioned female for 37 items, to the second-mentioned female for 28 items, to the first-mentioned male for 34 items, and to the second-mentioned male for 29 items. The slight unevenness in the number of items in each condition was compensated for by manipulating the proportion of references to first and second mentioned characters in the filler passages.
